# Supplementary material for: Traditional and Non-Traditional Clustering Techniques for Identifying Chrononutrition Patterns in University Students
Source: Nutrients. 2026 Jan 6;18(2):190. doi: 10.3390/nu18020190 (PMC12844766; doi:10.3390/nu18020190)
Supplement: Supplementary file 1 [file nutrients-18-00190-s001.zip › supplementary_materials-final.pdf]

Supplementary Materials

Traditional and Non-Traditional Clustering Techniques for Identifying Chrononutrition Patterns in University Students

Mora-Almanza et al.

Contents

1 Figure S1. PCA Biplot Composite (All Methods, k=2 to k=5) 2

2 Figure S2. Internal Validation Metrics (k=2 to k=5) 3

3 Table S1. Validation Metrics for k=2 to k=5 4

4 Figure S3. Gap Statistic Analysis 5

5 Table S2. Cluster Meal Timing Profiles (k=2 to k=5) 6

6 Figure S4. Silhouette Analysis Composite (k=2 to k=5) 8

7 Figure S5. Cross-Method Cluster Assignment Flow 9

8 Figure S6. Bootstrap Stability Analysis 10

1 Figure S1. PCA Biplot Composite (All Methods, k=2 to k=5)

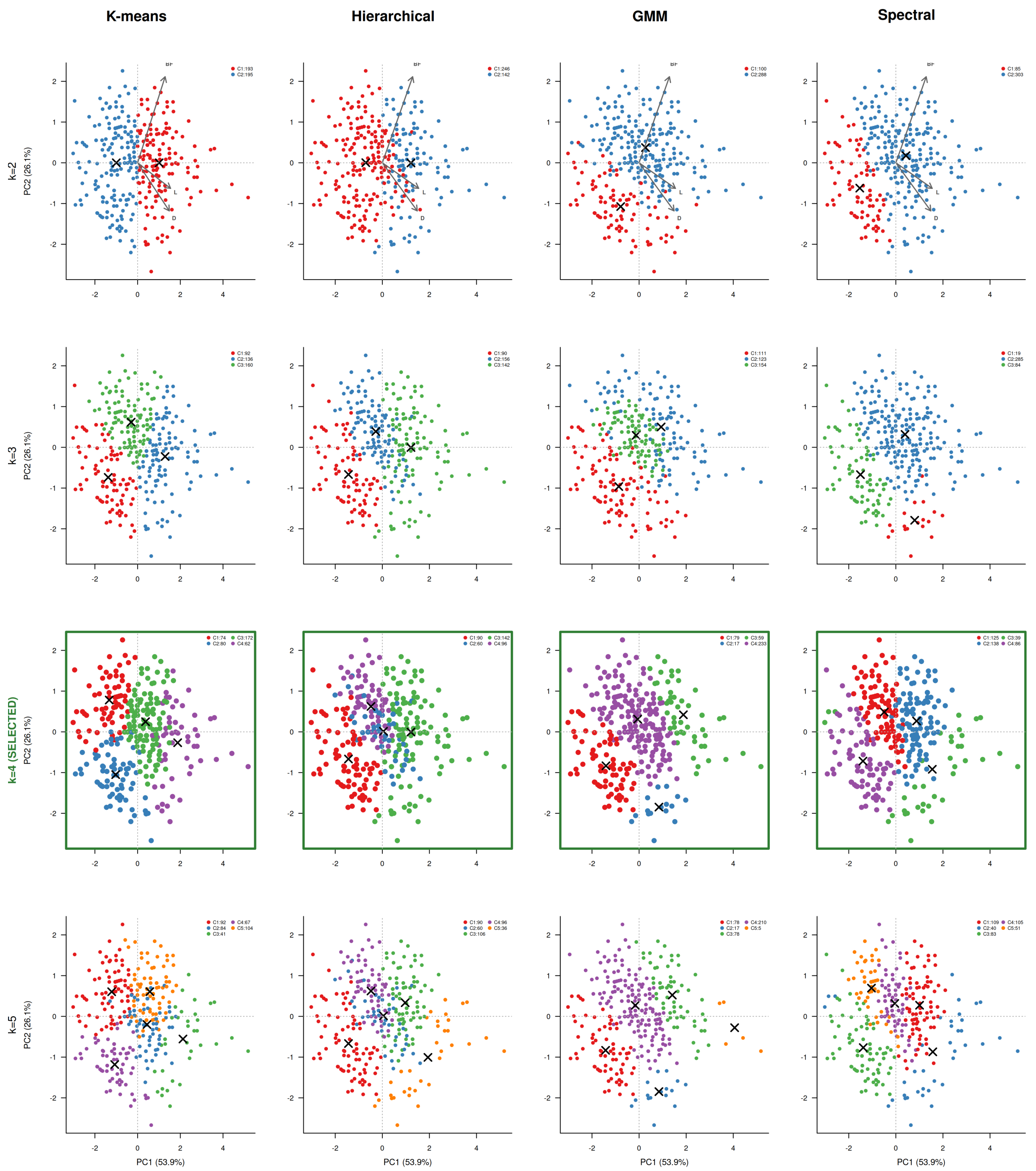

Figure S1: **Principal Component Analysis visualization of cluster solutions across meth-ods and k values.** Composite figure showing 16 panels organized as rows (k=2, k=3, k=4, k=5) and columns (K-means, Hierarchical, GMM, Spectral). Each panel displays participants projected onto the first two principal components (PC1 and PC2), colored by cluster assignment. Loading vectors indicate the contribution of each meal timing variable (Breakfast, Lunch, Din-ner) to the principal components. **The k=4 row (SELECTED) is highlighted** as the final solution, showing consistent cluster separation across all four methods. This visualization en-ables direct comparison of how cluster structure evolves with increasing k and how each method partitions the data space.

2 Figure S2. Internal Validation Metrics (k=2 to k=5)

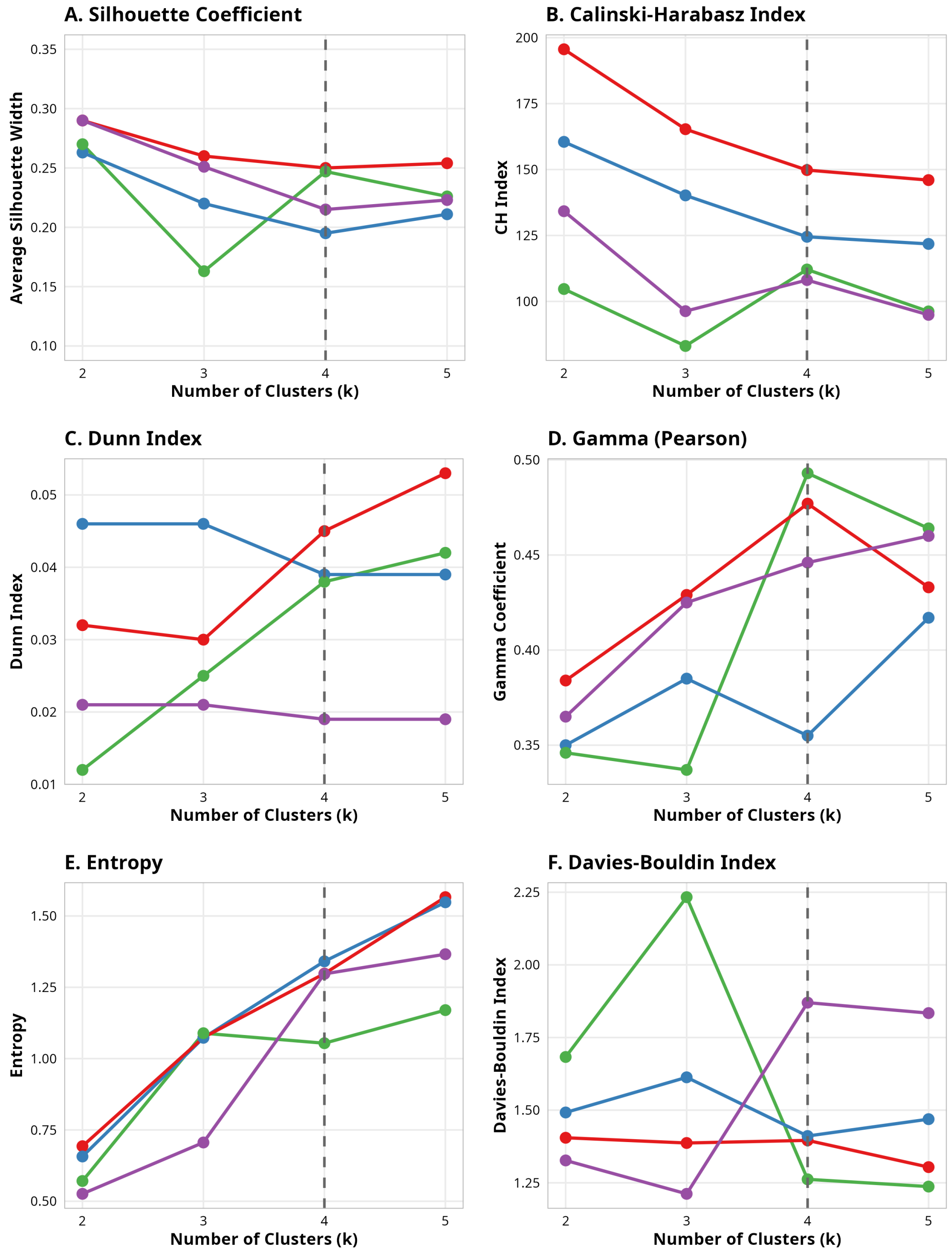

Dashed vertical line indicates selected k=4 solution. Note: Lower Davies-Bouldin values indicate better clustering.

Figure S2: **Internal validation metrics across cluster solutions (k=2 to k=5).** Six-panel comparison: (A) Silhouette coefficient (higher = better separation); (B) Calinski-Harabasz index (higher = better); (C) Dunn index (higher = better); (D) Gamma coefficient (higher = better);(E) Entropy (lower = more compact); (F) Davies-Bouldin index (lower = better clustering). Dashed line indicates selected k=4. Methods: K-means (red), Hierarchical (blue), GMM (green), Spectral (purple).

3 Table S1. Validation Metrics for k=2 to k=5

Table S1: **Internal validation metrics for cluster solutions k=2 to k=5 across four clustering methods.** Bold values indicate the best performance for each metric across all methods and k values. Higher values are better for Silhouette, Dunn, Calinski-Harabasz, and Gamma indices; lower values are better for Davies-Bouldin and Entropy. The k=4 solution was selected as it provided the best balance between metric performance and interpretability of identified patterns.

Method	k	Silhouette	Dunn	Calinski-Harabasz	Gamma	Davies-Bouldin	Entropy
K-means							
	2	**0.290**	0.032	**195.6**	0.384	1.405	0.693
	3	0.260	0.030	165.3	0.429	1.387	1.074
	**4**	0.250	0.045	149.8	0.477	1.396	1.298
	5	0.254	**0.053**	146.0	0.433	1.304	1.566
Hierarchical							
	2	0.263	0.046	160.5	0.350	1.492	0.657
	3	0.220	0.046	140.2	0.385	1.613	1.073
	**4**	0.195	0.039	124.5	0.355	1.411	1.341
	5	0.211	0.039	121.8	0.417	1.469	1.548
GMM							
	2	0.270	0.012	104.7	0.346	1.683	0.571
	3	0.163	0.025	83.1	0.337	2.233	1.089
	**4**	0.247	0.038	112.1	**0.493**	1.262	1.054
	5	0.226	0.042	96.2	0.464	**1.237**	1.170
Spectral							
	2	**0.290**	0.021	134.2	0.365	1.327	**0.526**
	3	0.251	0.021	96.3	0.425	**1.212**	0.706
	**4**	0.215	0.019	108.1	0.446	1.870	1.297
	5	0.223	0.019	94.9	0.460	1.834	1.366

Silhouette coefficient measures how similar an object is to its own cluster compared to other clusters (range: -1 to 1). Dunn index quantifies the ratio of minimum inter-cluster distance to maximum intra-cluster distance. Calinski-Harabasz index measures the ratio of between-cluster to within-cluster variance. Gamma (Hubert's) measures correlation between within-cluster and between-cluster distance rankings. Davies-Bouldin index measures the average similarity ratio of each cluster with its most similar cluster (lower values indicate better clustering). Entropy quantifies uncertainty in cluster assignments.

4 Figure S3. Gap Statistic Analysis

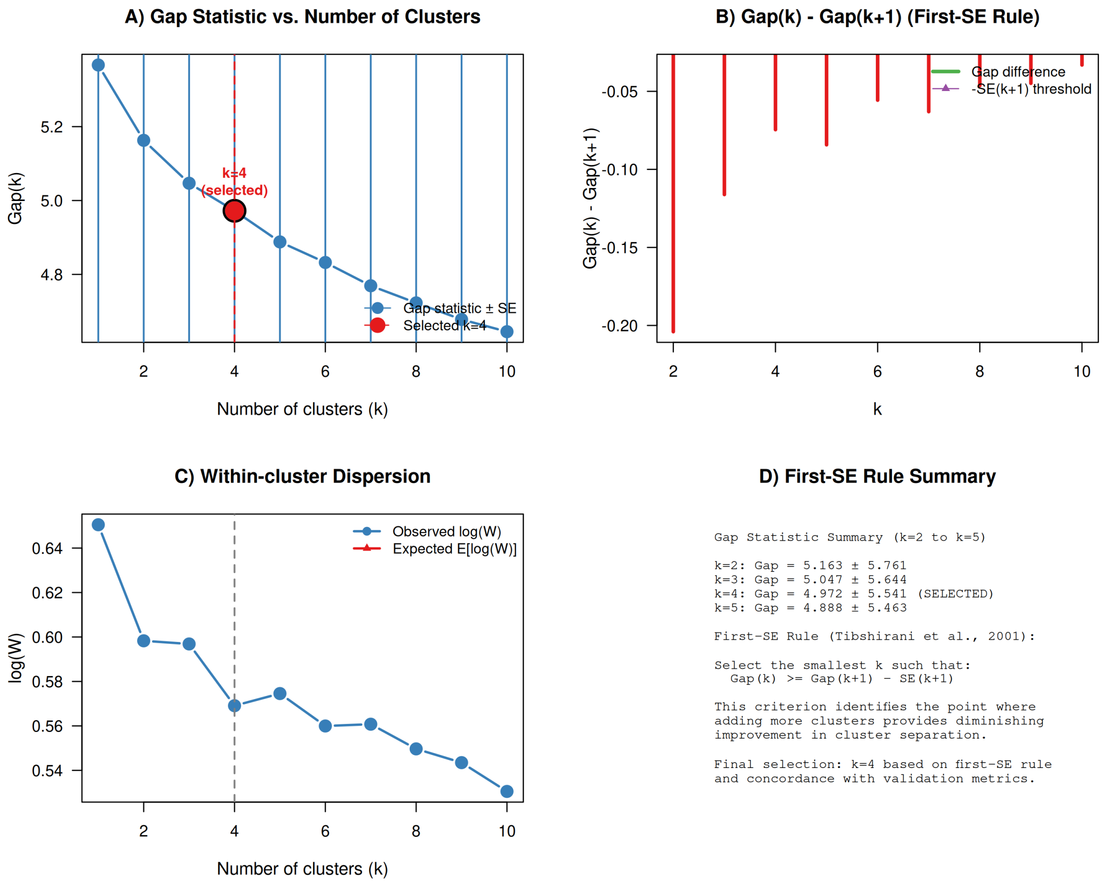

Figure S3: **Gap statistic analysis for optimal cluster number selection.** (A) Gap statistic values with standard error bars across k=1 to k=10. The Gap statistic compares the within-cluster dispersion to that expected under a uniform reference distribution. (B) Gap difference plot showing Gap(k) − Gap(k+1) with the standard error threshold for the first-SE r ule. According to the first-SE rule, the optimal k is the smallest value for which Gap(k) ≥ Gap(k+1) − SE(k+1). (C) Comparison of observed vs. expected log(W) values. (D) Summary of Gap statistic values for k=2 to k=5. The k=4 solution was selected based on concordance with other validation metrics, interpretability of the resulting clusters, and adequate stability.

5 Table S2. Cluster Meal Timing Profiles (k=2 to k=5)

Table S2: **Cluster meal timing profiles for k=2 to k=5 across all methods.** Values shown as median [Q1,Q3] in 24-hour format (HH:MM). Q1 and Q3 represent the 25th and 75th percentiles, respectively. The selected k=4 solution is highlighted in bold.

k	Method	Cluster	n	Breakfast	Lunch	Dinner
k=2						
2	K-means	C1	193	10:00 [09:00,10:30]	16:00 [15:30,16:30]	22:00 [21:30,22:30]
		C2	195	08:30 [07:00,09:00]	15:00 [14:30,15:30]	21:00 [20:22,21:15]
2	Hierarchical	C1	246	08:47 [07:00,09:00]	15:00 [14:30,15:30]	21:00 [20:30,21:30]
		C2	142	10:00 [09:00,10:30]	16:00 [16:00,17:00]	22:00 [21:30,22:30]
2	GMM	C1	100	06:43 [06:00,07:15]	15:30 [14:45,16:00]	21:00 [20:30,22:00]
		C2	288	09:30 [09:00,10:00]	15:30 [15:00,16:00]	21:30 [21:00,22:00]
2	Spectral	C1	303	09:30 [09:00,10:00]	15:30 [15:00,16:00]	21:30 [21:00,22:00]
		C2	85	06:30 [06:00,07:15]	15:00 [14:00,15:30]	20:40 [20:00,21:00]
k=3						
3	K-means	C1	92	06:35 [06:00,07:15]	15:00 [14:26,15:30]	21:00 [20:15,21:30]
		C2	161	09:15 [09:00,10:00]	15:00 [14:40,15:30]	21:00 [20:30,21:30]
		C3	135	09:40 [09:00,10:30]	16:00 [16:00,17:00]	22:00 [21:45,22:38]
3	Hierarchical	C1	90	06:45 [06:00,07:26]	15:00 [14:00,15:30]	20:52 [20:00,21:30]
		C2	156	09:00 [09:00,09:30]	15:00 [14:44,15:30]	21:00 [20:30,21:32]
		C3	142	10:00 [09:00,10:30]	16:00 [16:00,17:00]	22:00 [21:30,22:30]
3	GMM	C1	111	07:00 [06:00,07:30]	15:30 [14:43,16:00]	21:00 [20:30,22:00]
		C2	123	10:30 [09:30,11:00]	16:00 [15:30,17:00]	22:00 [21:00,22:30]
		C3	154	09:00 [09:00,09:30]	15:00 [15:00,15:56]	21:15 [21:00,21:56]
3	Spectral	C1	285	09:30 [09:00,10:00]	15:30 [15:00,16:00]	21:30 [21:00,22:00]
		C2	84	06:35 [06:00,07:04]	15:00 [14:00,15:30]	20:45 [20:00,21:15]
		C3	19	07:00 [06:13,07:30]	17:00 [16:52,17:30]	22:30 [22:00,22:38]
**k=4 (SELECTED)**						
**4**	**K-means**	**C1**	**94**	**09:00 [08:00,09:30]**	**14:30 [14:00,15:00]**	**20:30 [20:00,21:00]**
		**C2**	**68**	**06:15 [05:50,07:00]**	**15:30 [14:45,16:00]**	**21:00 [20:45,21:30]**
		**C3**	**59**	**10:30 [09:00,11:00]**	**17:00 [16:38,17:30]**	**22:30 [22:00,23:00]**
		**C4**	**167**	**09:30 [09:00,10:00]**	**15:30 [15:00,16:00]**	**21:30 [21:00,22:00]**
**4**	**Hierarchical**	**C1**	**90**	**06:45 [06:00,07:26]**	**15:00 [14:00,15:30]**	**20:52 [20:00,21:30]**
		**C2**	**60**	**09:00 [08:49,09:30]**	**15:00 [14:30,15:00]**	**22:00 [21:30,22:30]**
		**C3**	**142**	**10:00 [09:00,10:30]**	**16:00 [16:00,17:00]**	**22:00 [21:30,22:30]**
		**C4**	**96**	**09:00 [09:00,09:30]**	**15:00 [15:00,15:33]**	**21:00 [20:30,21:00]**
**4**	**GMM**	**C1**	**79**	**06:30 [06:00,07:00]**	**15:00 [14:30,15:30]**	**21:00 [20:22,21:30]**
		**C2**	**17**	**07:00 [06:15,07:30]**	**17:00 [17:00,17:30]**	**22:30 [22:00,22:45]**
		**C3**	**59**	**11:00 [10:30,11:30]**	**16:30 [16:00,17:15]**	**22:00 [21:30,22:52]**
		**C4**	**233**	**09:00 [09:00,10:00]**	**15:00 [15:00,16:00]**	**21:15 [21:00,22:00]**
**4**	**Spectral**	**C1**	**86**	**06:43 [06:00,07:26]**	**15:00 [14:04,15:30]**	**21:00 [20:15,21:30]**
		**C2**	**39**	**08:00 [07:00,10:30]**	**17:30 [17:00,17:52]**	**22:30 [22:00,23:00]**
		**C3**	**138**	**10:00 [09:00,10:30]**	**16:00 [15:30,16:30]**	**22:00 [21:00,22:00]**
		**C4**	**125**	**09:00 [08:45,09:30]**	**15:00 [14:30,15:00]**	**21:00 [20:30,21:30]**

Continued on next page

Table S2 – *Continued from previous page*

k	Method	Cluster	n	Breakfast	Lunch	Dinner
k=5						
5	K-means	C1	41	10:00 [08:30,11:00]	17:15 [17:00,17:30]	22:30 [22:00,23:00]
		C2	84	09:00 [08:30,09:30]	15:00 [15:00,15:30]	22:00 [22:00,22:30]
		C3	92	09:00 [08:00,09:30]	14:30 [14:00,15:00]	20:30 [20:00,21:00]
		C4	67	06:15 [05:50,07:00]	15:30 [14:45,16:00]	21:00 [20:45,21:30]
		C5	104	10:00 [09:30,10:30]	16:00 [15:30,16:19]	21:00 [21:00,21:30]
5	Hierarchical	C1	90	06:45 [06:00,07:26]	15:00 [14:00,15:30]	20:52 [20:00,21:30]
		C2	60	09:00 [08:49,09:30]	15:00 [14:30,15:00]	22:00 [21:30,22:30]
		C3	106	10:00 [09:30,10:30]	16:00 [16:00,16:30]	22:00 [21:04,22:00]
		C4	96	09:00 [09:00,09:30]	15:00 [15:00,15:33]	21:00 [20:30,21:00]
		C5	36	09:15 [07:08,10:38]	17:30 [17:00,18:00]	22:30 [22:00,23:00]
5	GMM	C1	78	06:30 [06:00,07:00]	15:00 [14:30,15:30]	21:00 [20:19,21:30]
		C2	17	07:00 [06:15,07:30]	17:00 [17:00,17:30]	22:30 [22:00,22:45]
		C3	78	10:30 [10:00,11:00]	16:00 [16:00,17:00]	22:00 [21:00,22:30]
		C4	210	09:00 [08:45,09:30]	15:00 [14:56,15:45]	21:15 [21:00,22:00]
		C5	5	12:00 [11:30,12:00]	18:00 [18:00,19:00]	24:00 [23:30,24:00]
5	Spectral	C1	86	06:43 [06:00,07:26]	15:00 [14:04,15:30]	21:00 [20:15,21:30]
		C2	18	07:05 [06:19,07:30]	17:08 [17:00,17:30]	22:30 [22:00,22:41]
		C3	154	10:00 [09:00,10:30]	16:00 [15:30,16:00]	22:00 [21:00,22:00]
		C4	23	10:30 [10:00,11:30]	17:30 [17:00,18:00]	22:45 [22:00,23:45]
		C5	107	09:00 [08:38,09:30]	15:00 [14:30,15:00]	21:00 [20:30,21:30]

Note: Values are median [Q1,Q3] where Q1 = 25th percentile and Q3 = 75th percentile. Clustering was performed on z-score standardized data (seed = 2025, K-means nstart = 50). n = number of participants assigned to each cluster. Times are shown in 24-hour format (HH:MM).

6 Figure S4. Silhouette Analysis Composite (k=2 to k=5)

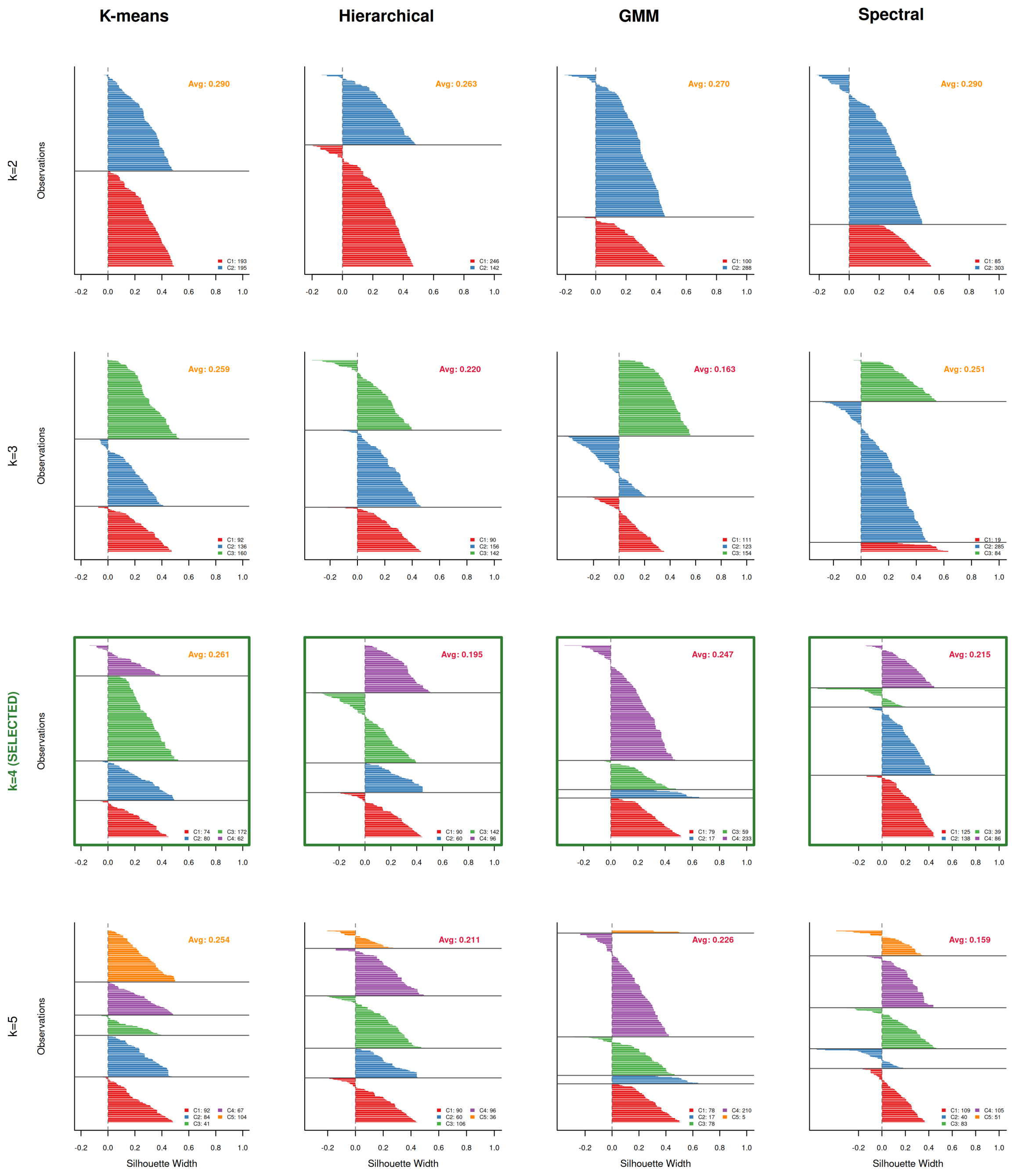

Figure S4: **Silhouette analysis across all k values and clustering methods.** Composite figure showing 16 panels organized as rows (k=2, k=3, k=4, k=5) and columns (K-means, Hierarchical, GMM, Spectral). Each panel displays the silhouette profile for one combination of k and method, with horizontal bars representing individual participants colored by cluster assignment. Bar width indicates the silhouette coefficient, measuring how similar a participant is to their own cluster compared to other clusters. Values range from -1 (poor fit) to 1 (excellent fit). The average silhouette width is shown for each panel, with color coding indicating quality: green (>0.40), orange (0.25–0.40), and red (<0.25). **The k=4 row (SELECTED) is highlighted**, showing that K-means achieved the highest mean silhouette (0.261), followed by GMM (0.247), Spectral (0.215), and Hierarchical (0.195). This visualization enables direct comparison of cluster quality across all solutions.

7 Figure S5. Cross-Method Cluster Assignment Flow

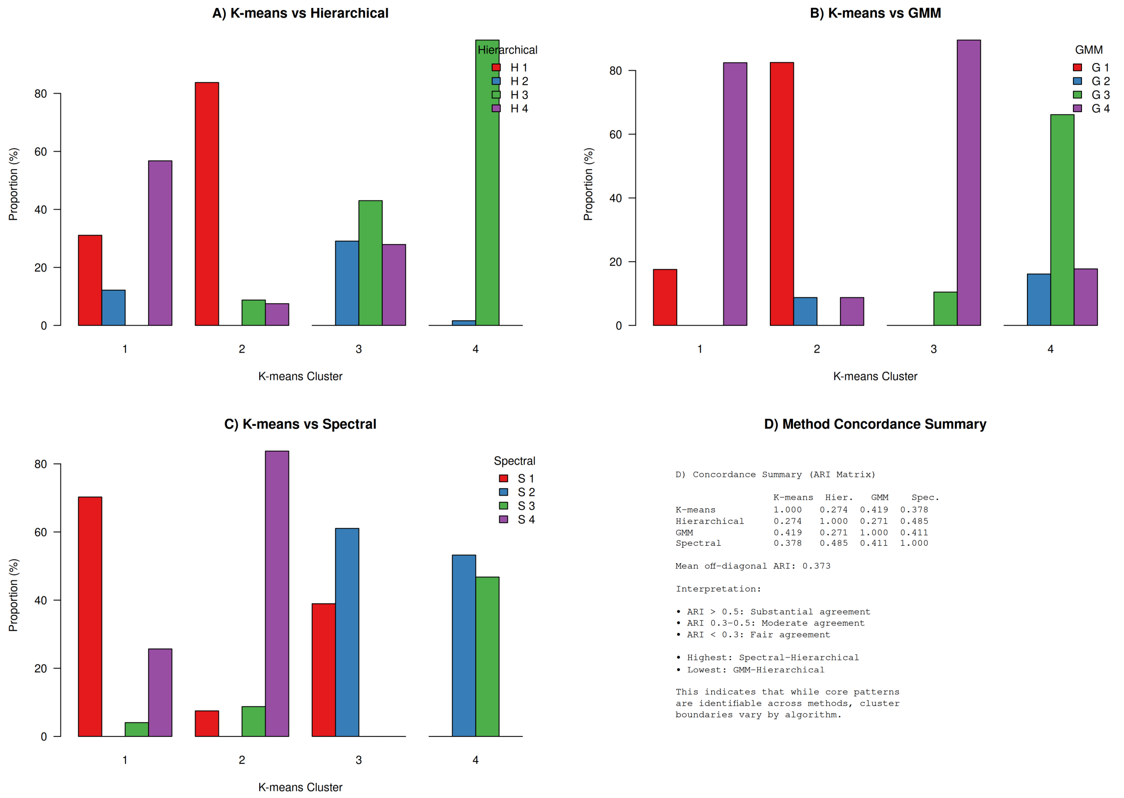

Figure S5: **Cross-method concordance in cluster assignments for k=4.** (A–C) Stacked bar charts showing how participants assigned to each K-means cluster are distributed across clusters in other methods (Hierarchical, GMM, Spectral). (D) Summary of cross-method agreement patterns. The visualization reveals that while core chrononutrition patterns are identifiable across methods, cluster boundaries are partially method-dependent. Clusters with consistent membership across methods (shown by dominant colors in each bar) represent the most robust chrononutrition patterns, while mixed coloring indicates boundary cases where method choice influences classification.

8 Figure S6. Bootstrap Stability Analysis

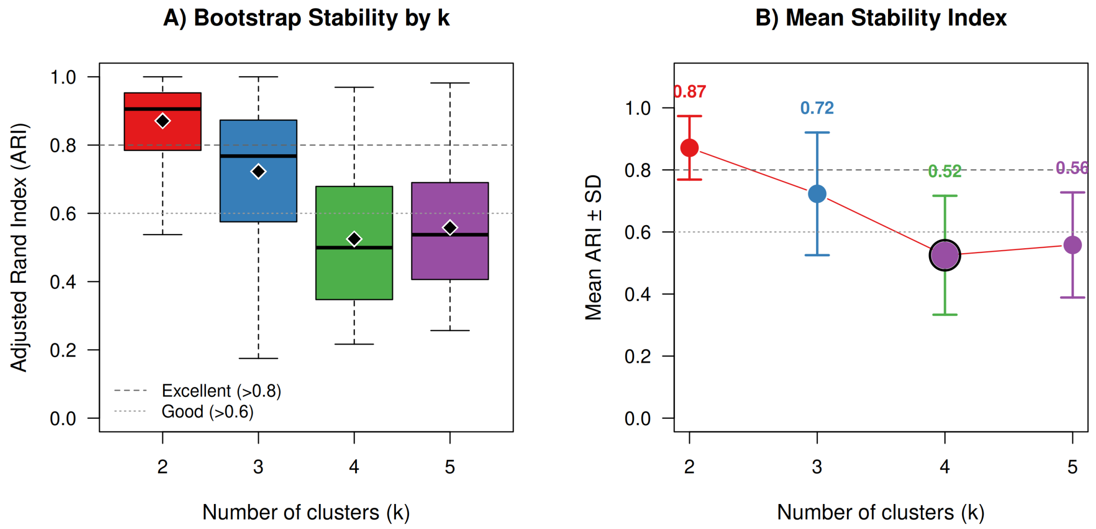

Figure S6: **Bootstrap stability analysis for cluster solutions k=2 to k=5.** (A) Boxplot showing the distribution of Adjusted Rand Index (ARI) values across 500 bootstrap resamples for each k value. Higher ARI indicates greater stability of cluster assignments when data is resampled with replacement. (B) Mean stability index with standard deviation error bars. The dashed lines indicate thresholds for excellent (>0.8) and good (>0.6) stability. Results demonstrate moderate to good stability across all k values, with k=4 showing consistent performance across bootstrap samples. This analysis confirms that the selected k=4 solution produces reproducible cluster assignments.

Summary of Supplementary Materials

This supplementary document provides comprehensive methodological details and validation results for the chrononutrition clustering analysis. The materials are organized to demonstrate the systematic process of selecting the optimal number of clusters (k=4) from four candidate solutions (k=2 to k=5) across four clustering methods (K-means, Hierarchical with Ward's linkage, Gaussian Mixture Models, and Spectral clustering).

Figure S1: PCA Visualization

- **Figure S1** presents a 16-panel composite visualization showing PCA biplots for all combinations of k values (rows: k=2, k=3, k=4, k=5) and methods (columns: K-means, Hierarchical, GMM, Spectral). This comprehensive view enables direct comparison of how cluster structure evolves with increasing k and demonstrates the consistency of the k=4 solution across methods.

Figure S2, Table S1, Figure S3: Cluster Number Selection

- **Figure S2** presents six key internal validation metrics (Silhouette, Calinski-Harabasz, Dunn, Gamma, Entropy, and Davies-Bouldin) across k=2 to k=5 for all four clustering methods, demonstrating that k=4 provides optimal balance between cluster separation and parsimony.
- **Table S1** provides comprehensive validation metrics for all combinations of k values and methods, including the Davies-Bouldin index which measures the average similarity ratio of each cluster with its most similar cluster (lower values indicate better clustering).
- **Figure S3** shows Gap statistic analysis with the first-SE rule, providing additional evidence for the k-selection process.

Table S2: Cluster Profiles

- **Table S2** provides meal timing profiles (breakfast, lunch, dinner times) for each cluster across all k values and methods. Values are shown as median [Q1,Q3], where Q1 and Q3 represent the 25th and 75th percentiles, respectively. This non-parametric representation provides robust characterization of the temporal eating patterns within each cluster solution.

Figures S4–S6: Validation and Stability

- **Figure S4** presents a 16-panel composite visualization showing silhouette analysis for all combinations of k values (rows: k=2, k=3, k=4, k=5) and methods (columns: K-means, Hierarchical, GMM, Spectral), enabling direct comparison of cluster quality across all solutions and confirming that K-means achieves the highest mean silhouette for k=4.
- **Figure S5** visualizes cross-method concordance through participant flow diagrams, showing how cluster assignments compare across methods.
- **Figure S6** presents bootstrap stability analysis, demonstrating that the k=4 solution produces reproducible cluster assignments across 500 bootstrap resamples.

Together, these materials support the methodological rigor of the main analysis by providing transparency regarding the systematic evaluation of cluster solutions from k=2 to k=5, the selection criteria for k=4, and the validation of this solution across multiple methods.
